# Storage of Maize in Purdue Improved Crop Storage (PICS) Bags

**DOI:** 10.1371/journal.pone.0168624

**Published:** 2017-01-10

**Authors:** Scott B. Williams, Larry L. Murdock, Dieudonne Baributsa

**Affiliations:** Department of Entomology, Purdue University, West Lafayette, Indiana, United States of America; Henan Agricultural University, CHINA

## Abstract

Interest in using hermetic technologies as a pest management solution for stored grain has risen in recent years. One hermetic approach, Purdue Improved Crop Storage (PICS) bags, has proven successful in controlling the postharvest pests of cowpea. This success encouraged farmers to use of PICS bags for storing other crops including maize. To assess whether maize can be safely stored in PICS bags without loss of quality, we carried out laboratory studies of maize grain infested with *Sitophilus zeamais* (Motshulsky) and stored in PICS triple bags or in woven polypropylene bags. Over an eight month observation period, temperatures in the bags correlated with ambient temperature for all treatments. Relative humidity inside PICS bags remained constant over this period despite the large changes that occurred in the surrounding environment. Relative humidity in the woven bags followed ambient humidity closely. PICS bags containing *S*. *zeamais*-infested grain saw a significant decline in oxygen compared to the other treatments. Grain moisture content declined in woven bags, but remained high in PICS bags. Seed germination was not significantly affected over the first six months in all treatments, but declined after eight months of storage when infested grain was held in woven bags. Relative damage was low across treatments and not significantly different between treatments. Overall, maize showed no signs of deterioration in PICS bags versus the woven bags and PICS bags were superior to woven bags in terms of specific metrics of grain quality.

## Introduction

Maize is one of the most important cereal grains grown worldwide and it is the basis for a significant portion of Sub-Saharan Africa’s diet. The cultivation of maize in Africa has increased, eclipsing traditional grains like millet and sorghum [1], but its production is still constrained by a number of factors. Insect pests of stored maize like *Sitophilus zeamais* (Motschulsky) are capable of inflicting serious damage to it after harvest, resulting in 20–50% loss after 3–6 months and even total loss under worst-case conditions [2–5].

Prevention of pests is important as losses during storage reduce food availability, quality, and the stability of farmers’ food supply and income [4]. Options to prevent insect damage are fewer for smallholder farmers in Africa than for their counterparts elsewhere. Synthetic pesticides are expensive, may not be available in the market regularly, and may be illegally blended with other compounds [6, 7]. Even when applied properly, damage can occur and the need to repeatedly apply chemical agents increases the chance of human or environmental toxicity [8, 9]. Synthetic pesticides are a non-preferred option for most smallholder farmers seeking to control grain pests.

The Purdue Improved Crop Storage (PICS) bag has proven to be an effective alternative to chemical pesticides for stored grain. This bag uses two liners of high-density polyethylene (HDPE) and an outer layer composed of woven polypropylene. Together, they create low-oxygen environments that reduce insect development [10]. As much as 98% of all insect pests can be eliminated within just 1 month of storage, reducing damage and weight loss caused by feeding [11].

PICS bags have been successfully demonstrated to protect cowpea against its most common pest, the cowpea bruchid, *Callosobruchus maculatus* (F.). Close to 50% of the cowpea not sold at harvest in West Africa is now stored in simple hermetic containers [12–14]. The success of PICS bags with cowpea encourage industrious farmers to store other types of grain, like maize, in PICS bags. Bauoa et al. [11] demonstrated that PICS bags protect maize against insect pests during field trials with no loss of quality over 6 months. To confirm Bauoa et al.’s observations, we performed additional trials under laboratory-controlled conditions to provide additional support for the use of PICS bags to store maize.

## Methods

### Experimental setup

**Fig 1:**
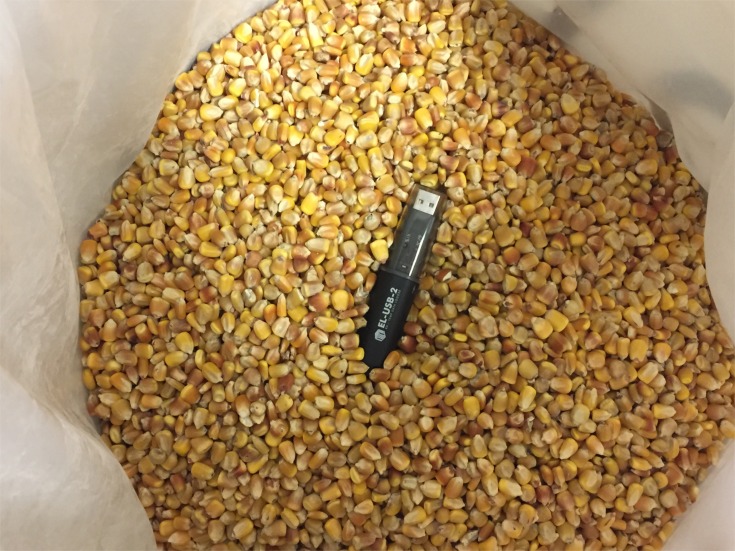
Data logger as seen before sealing of the PICS bag. Wireless data loggers were used to measure temperature and relative humidity conditions within PICS bags. Data loggers were pushed into the center of the maize bulk prior to sealing the bags.

Yellow maize grain (Yellow Trucker’s Favorite, Lot#502) was purchased from the Wax Seed Co. (Armory, Alabama). A total of 320 kg of grain was stored as 20 kg units in sixteen bags. Bags were stored in a sealed room on the outside corner of Whistler Hall at Purdue University (West Lafayette, IN) for 8 months between May 2014 and January 2015. Eight units were stored in hermetically-sealed PICS triple bags and another eight were stored in woven mesh bags as a control. One kilogram of maize, infested with *S*. *zeamais*, was added to the top layer of four bags from each treatment group prior to closing. The remaining bags were left non-infested. Wireless data loggers (EL-USB-2, Lascar, Erie, PA, United States) were put into the bags to measure the internal temperature and relative humidity every 12 h (Fig 1). Additional data loggers were kept inside the storage room for comparison. Each bag was tied and sealed and placed in a quarantine room that featured a double-layer of plastic sheeting to prevent escaping insects inside the enclosure for the duration of the study. A separate room, located in the central portion of Whistler Hall was used to store woven bags filled with non-infested maize. This arrangement prevented possible contamination of non-infested bags by individual *S*. *zeamais* escaping from infested woven bags.

### Sample collection

Data was collected every two months. At each interval, 600 mL of cowpea were removed from each bag. These samples were collected as 20, 30 mL subsamples, randomly collected using a probe. All samples were stored in individual glass Mason jars and stored in a freezer until needed. The following data were collected: 1) internal oxygen level, 2) grain moisture content, 3) percent germination rate, and 4) relative damage as described below.

### Oxygen readings

Internal oxygen was measured using an Oxysense 5250i® oxygen reader (Oxysense, Dallas, TX). The Oxysense uses a combination of fluorescent yellow dots and an ultraviolet light pen to detect oxygen levels. The dots can be placed on the inside of any transparent container prior to it being closed. Once the dot is placed, the light pen shines ultraviolet light onto its surface that excites the fluorescent pigment of the yellow dot. This excitation of the pigment causes the dot to “flash”. Oxygen “quenches” this reaction, so in environments with normal atmospheric oxygen, the dot only emits a few flashes. As oxygen declines in the environment, there is less oxygen to suppress the yellow dot’s reaction to the UV light and the oxysense records more flashes. The Oxysense can then translate the number of flashes it records into an estimate of the percent oxygen level within the container.

Two oxydots were used per bag, each bonded to a plastic petri dish and then attached with tape to the inner walls of both liners for each PICS bag. A small window was cut from the woven mesh layer of each PICS bag so that the oxydots were visible. Petri dishes were attached directly to the woven mesh layer in control bags. Each of the mesh bags had a small hole cut into the material just large enough to take readings with the oxysense reader.

Oxygen data was collected at regular intervals throughout the treatment period. Measurements were at first taken multiple times a day and then the interval was gradually increased with time, with readings eventually becoming separated by one week. Oxygen data was collected over the entire trial period.

### Moisture content

Grain moisture was measured using a DICKEY-JOHN (Auburn, IL) mini GAC® plus Grain Moisture tester. After first taking a blank reading of the empty container, the unit was filled with approximately 400 mL of maize. Any excess at the top was brushed off and the moisture measurements then taken. Moisture level was recorded as the percentage of the total grain mass.

### Relative damage

Each 600 mL pooled grain sample had four, 50 mL subsamples removed to evaluate the level of grain damage. The number of damaged and undamaged kernels in each subsample were visually counted and separated. Subsamples of each were dried to 0% moisture by heating in an oven at 60°C for 5 days. The dry weight of the damaged and undamaged grain for each subsample was then determined. Relative percent damage was calculated using the following equation [15]:

$X_{rel}=\left[\frac{\left(W_{u}*N_{d}\right)-\left(W_{d}*N_{u}\right)}{W_{u}*(N_{u}+N_{d})}\right]*100$ (1) N_d_ = Number of damaged grainsN_u_ = Number undamaged grains,W_u_ = Dry weight of undamaged grains,W_d_ = Dry weight of damaged grains

This equation compares the number of damaged and undamaged grains based on their weighted proportions. Using combined physical grain damage and weight takes into account both visible insect damage and hidden damage caused by insect larvae feeding inside the grain. The equation is an effective alternative to estimate grain damage that avoids collecting and weighing dust generated by insect feeding [15].

### Germination rate

Two samples of 50 seeds were removed from each bag sample. The grain was bathed in a 10% bleach solution for two minutes and then rinsed three times with water. Each sample of grain was wrapped with wet paper towels and stored at ambient room temperature (~24°C) in a dark drawer. After one week, the samples were removed and the number of grains with at least part of the radicle breaking through the seed coat was counted as germinated. Data were recorded as a percentage of the number of germinated kernels out of the total number of kernels sampled.

### Analysis

Temperature and humidity readings from data loggers kept within PICS bags were compared against ambient environmental conditions using Pearson’s correlation. Between-treatment effects on average oxygen level were compared using Analysis of Variance (ANOVA). Differences in oxygen levels between the inter-liner space and the inner liner were assessed using a paired t-test. Effects of treatment conditions on grain moisture, relative damage, and germination rate were also compared using ANOVA. Post-hoc comparisons were made between groups using Fisher’s exact test where applicable. Significant values were reported at the α = 0.05 level unless noted otherwise.

## Results

### Temperature and humidity

**Fig 2:**
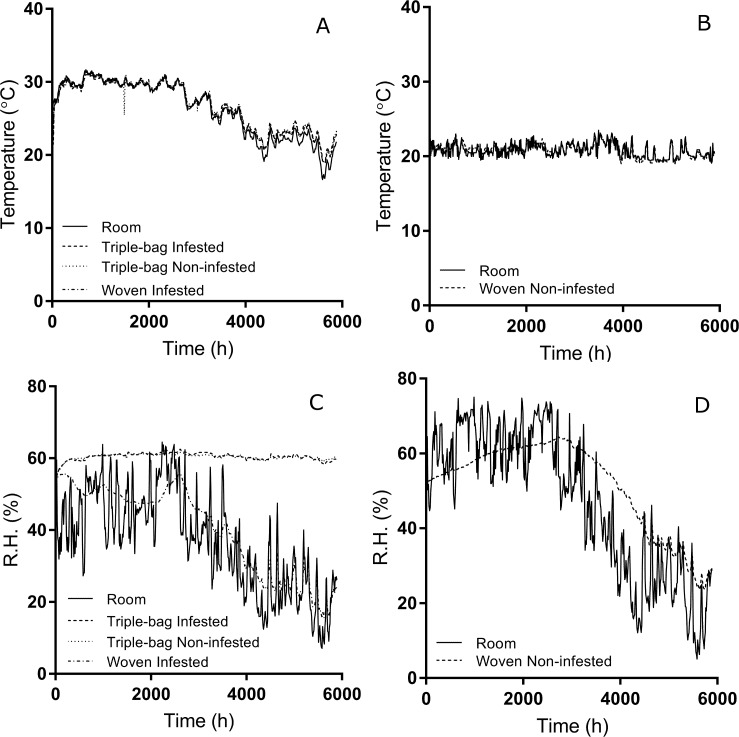
Temperature and Relative Humidity (RH) readings from two study locations. Temperature readings in both study locations (A, B) (see Methods and Materials) ranged between 20 and 30°C. Temperatures varied by about 14 degrees in the first location, while the second location’s temperature varied by about 4 degrees. Temperatures within the treatment bags correlated closely with environmental temperature. The R.H. of both rooms (C, D) were similar over the 8 month period. The R.H. in woven bags correlated with the environmental R.H. The R.H. within triple bags, however, did not change with environmental humidity and remained within a narrow range for the duration of the trial.

Internal temperatures of all bags stored in the primary quarantine room correlated closely with environmental temperature (Table 1) (Fig 2A and 2B). The association between non-infested woven bags’ temperature and ambient temperature was weaker, but still present. Relative humidity in both woven bag treatment groups also correlated well with environmental conditions (Table 2) (Fig 2C and 2D). Relative humidity in both triple bag treatments remained constant for the entire testing period and the association between the R.H. of the infested (Pearson’s correlation, *P* = 0.42) and non-infested triple bags (Pearson’s correlation, *P* = 0.37) and room R.H. was much lower than what we observed in the woven bags.

**Table 1:** Results of Pearson’s correlation comparing treatment bag temperature to room temperature.

Treatment	Room	df	Pearson's correlation
Infested Triple Bags	1	492	0.99
Non-infested Triple Bag	1	492	0.99
Infested Woven Bag	1	492	0.99
Non-infested Woven Bag	2	492	0.72

**Table 2:** Results of Pearson’s correlation comparing bag relative humidity to room RH.

Treatment	Room	df	Pearson's correlation
Infested Triple Bags	1	492	0.42
Non-infested Triple Bag	1	492	0.37
Infested Woven Bag	1	492	0.84
Non-infested Woven Bag	2	492	0.84

### Internal oxygen levels

**Fig 3:**
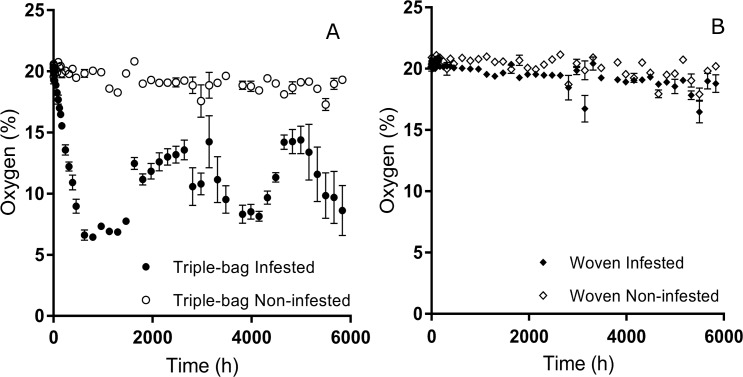
Oxygen levels observed in sealed triple bags and unsealed woven bags over an eight month period. Oxygen levels in infested triple bags (black circles) declined rapidly during the first month of the study period. Opening the bags allowed for fresh oxygen to enter and raise the overall percent oxygen, but these values remained lower than the levels recorded for the non-infested triple bags (white circles), infested woven bags (black diamond), and non-infested woven bags (white diamond) and would gradually decline as surviving insects continued to deplete the oxygen inside.

We observed different average levels of oxygen between our four treatment groups throughout the study (*F* = 456.52; d.f. = 3,767; *P* < 0.001) (Fig 3). Infested PICS bags had internal oxygen levels that were substantially lower than the other three treatment groups throughout the eight month time period. Even when bags were opened briefly at each sampling period, infested PICS bags continued to have lower levels of oxygen than the other treatment groups. There was no substantial difference in oxygen levels between the other treatment groups.

We observed significant differences in the oxygen levels recorded within the innermost liner and the space between the inner and middle liners for infested PICS bags (*t* = -10.43; d.f. = 312; *P* < 0.001). Oxygen levels within the innermost liner were on average 3.82 ± 0.36% lower than levels observed between the liners. This difference in oxygen levels could be as great as 8.16% in some cases. There was no significant difference in the oxygen levels between middle and inner liners for non-infested PICS bags, (*t* = -0.84, d.f. = 282; *P* = 0.404).

### Grain moisture

**Fig 4:**
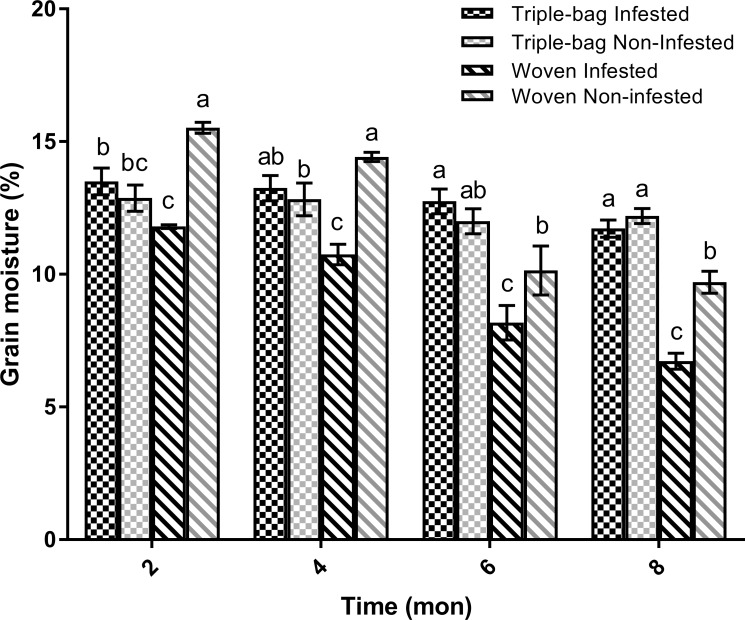
Grain moisture in sealed triple bags and unsealed woven bags over eight months. Both triple bag treatments remained relatively stable during the eight month study period, staying approximately between 12 and 13%. Grain moisture declined in both woven bag treatment groups, with the infested group losing 5.1% and the non-infested bags losing an average of 5.8% moisture content.

Storing maize in either triple or woven bags had a significant effect on grain moisture over time (Fig 4). The moisture content of maize stored in triple bags was consistent throughout the course of the study (Table 3). Maize stored in woven bags lost significant amounts of moisture across the study period and was much drier than maize stored in triple bags. This was true regardless of whether the bags were infested or not. Overall, maize stored in woven bags lost 5% of their total grain moisture between 4 and 8 months. Grain moisture in both triple bag treatments, in contrast, remained relatively constant across the entire study period, losing only 1–2% moisture over 8 months, which was not a statistically-significant change.

**Table 3:** Results of ANOVA analyses comparing the treatment effect on grain moisture.

Sampling Period	Treatment	Grain Moisture (%)^†^	DF	F-value	P-value
Initial	All Grain	11.7 ± 0.1			
2 months	TBI	13.5 ± 0.5**	3, 15	17.96	X < 0.001
	TBN	12.9 ± 0.5**			
	WI	11.8 ± 0.071***			
	WN	15.53 ± 0.21*			
4 months	TBI	13.25 ± 0.48**	3, 15	11.97	X = 0.001
	TBN	12.83 ± 0.61**			
	WI	10.75 ± 0.39***			
	WN	14.43 ± 0.17*			
6 months	TBI	12.75 ± 0.46*	3, 15	9.8	X = 0.002
	TBN	12.0 ± 0.47*			
	WI	8.18 ± 0.65***			
	WN	10.15 ± 0.92**			
8 months	TBI	11.73 ± 0.33*	3, 15	55.16	X < 0.001
	TBN	12.20 ± 0.29*			
	WI	6.73 ± 0.3***			
	WN	9.7 ± 0.41**			

^†^Initial moisture level for stored maize was 11.7 ± 0.1% before it was put into treatment bags.

*Asterisks indicate significant differences in grain moisture (%) between our four treatment groups. Treatments with the same number of asterisks are statistically similar and ones with different numbers of stars are statistically different.

### Relative damage

**Fig 5:**
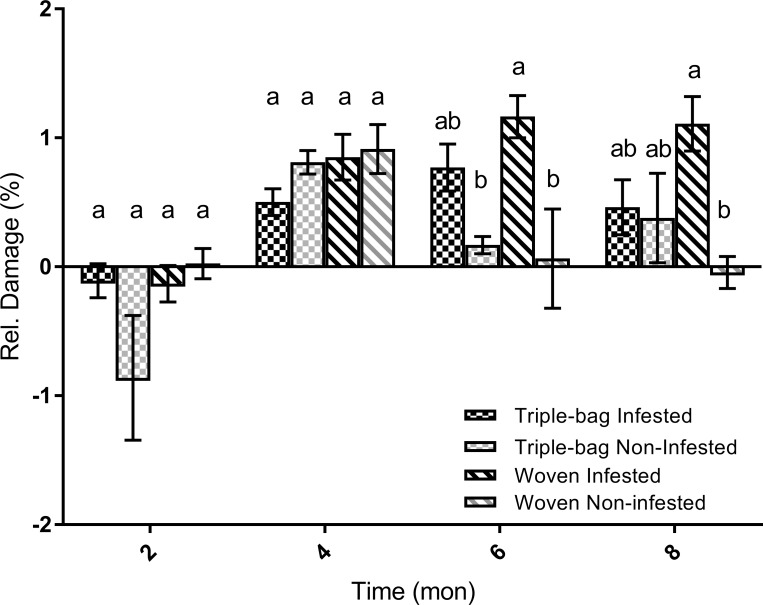
Relative damage observed in stored maize over eight months. Damage to the maize varied from month to month. Recorded levels of grain damage were highly variable from one time point to the next. Only the woven, infested group (light gray, stripes) had a consistent increase in grain damage over the course of 8 months.

Insect damage to the stored maize varied greatly with time (Fig 5) and we found no consistency in terms of the level of damage in response to the treatment conditions (Table 4). Damage levels showed no trends over time and there was high variability among the samples. Due to the lack of any clear statistical trends or relationships, we are hesitant to make any sort of hard conclusions.

**Table 4:** Results of ANOVA analyses comparing treatment effect of relative damage to maize grain.

Treatment	Treatment	Rel. Damage (%)	DF	F-value	P-value
2 months	TBI	-0.11 ± 0.12*	3, 63	F = 2.26	X = 0.091
	TBN	-0.86 ± 0.96*			
	WI	-0.13 ± 0.18*			
	WN	0.02 ± 0.08*			
4 months	TBI	0.5 ± 0.2*	3, 63	F = 1.54	X = 0.214
	TBN	0.81 ± 0.13*			
	WI	0.85 ± 0.26*			
	WN	0.91 ± 0.3*			
6 months	TBI	0.77 ± 0.38*	3, 63	F = 8.28	X < 0.001
	TBN	0.17 ± 0.1**			
	WI	1.16 ± 0.37*			
	WN	0.06 ± 0.34*			
8 months	TBI	0.46 ± 0.49*	3, 63	F = 4.04	X = 0.011
	TBN	0.38 ± 0.63*			
	WI	1.11 ± 0.44*			
	WN	-0.044 ± 0.15**			

*Asterisks indicate significant differences in grain moisture (%) between our four treatment groups. Treatments with the same number of asterisks are statistically similar and ones with different numbers of stars are statistically different.

### Germination

**Fig 6:**
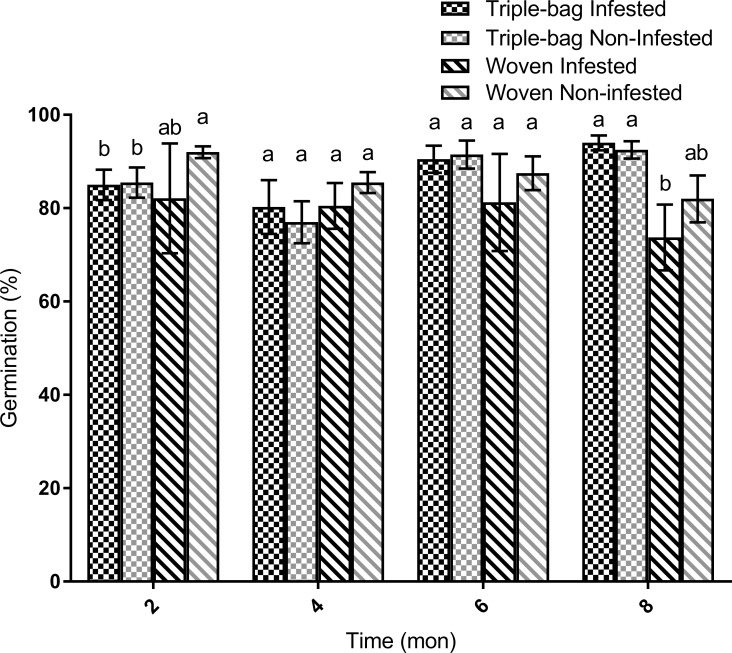
Maize germination rate after eight months of storage. There was no significant effect of storage treatment on germination during the first 6 months of the trial. After 8 months of storage, grain stored in infested woven bags had significantly lower germination rates than those stored in triple bags and non-infested woven bags.

Overall, germination rates for maize stored in triple bags were almost equal to rates observed in non-infested controls. Maize germination rates ranged between 70 and 95% across our eight month study period (Fig 6). Germination of maize stored in triple bags was lower compared to rates observed in woven, non-infested bags after two months (*F* = 3.28, d.f. = 3, 31, *P* = 0.036). However, these differences disappeared by the four month mark (*F* = 0.6, d.f. = 3, 31, *P* = 0.62) and continued for the remainder of the trial (*F* = 0.61, d.f. = 3, 31, *P* = 0.61). After 8 months, germination rates for maize stored in the infested woven bags were statistically lower than rates observed in non-infested woven bags (9% lower) and all triple bags (30% lower) (*F* = 4.41, d.f. = 3, 31, *P* = 0.011).

## Discussion

The quality of maize grain stored in PICS bags was either equal to or superior to the quality of maize stored in the woven, polypropylene bags over the eight month storage period. Whether clean or infested with *S*. *zeamais*, maize in PICS triple bags remained consistent for benchmarks of quality, such as stable moisture content and germination. This was not the case for maize stored in woven bags, especially those infested by the weevil. These results are consistent with the results of other studies focused on PICS bags, which demonstrated better outcomes for grain stored in the triple bags than for grain stored in other ways [10, 11, 16–21].

PICS triple bags possess several properties that allow them to maintain grain quality. First, the plastic liners greatly hinder transmission of oxygen into and out of the grain bulk. When oxygen levels fall rapidly in the infested PICS bag group, this creates a negative feedback loop on the insect population that was driving this decline. Consumption of oxygen by the insects led to conditions that were unfavorable for further population growth. Once oxygen levels fell to around 5%, oxygen, consumption noticeably slowed down and feeding activity ceased. Even after the bags were opened and resealed during the bimonthly sample collection, the surviving insects drove the oxygen below sustainable levels again.

Murdock et al. [19] have pointed out that the PICS bags’ ability to create low-oxygen environments is the key to their protective nature. Contributing to this protection is the higher level of oxygen within the space between the two polyethylene liners. While a plastic membrane like the polyethylene liners can permit minimal diffusion of oxygen, this process is slow and dependent on the difference in concentrations of oxygen on either side of the membrane. Our current results show for the first time that oxygen levels in the inter-liner space can be 3–4% higher than the inner grain environment. Thus, as originally suggested [20] this space of higher oxygen creates a buffer zone that discourages oxygen movement across both liners, as it reduces the difference in oxygen concentration on either side. The result is slower movement of the oxygen into the grain environment from the ambient air than if there were a single layer, even if that layer were thicker than typical for PICS bags. This fact establishes the value of the double-layer of HDPE as part of the triple bag configuration.

Preventing water vapor transmission is another valuable trait of triple layer bags. PICS bags had higher internal R.H. and maintained grain moisture better than woven bags, in which the grain dried out as ambient R.H. dropped over the 8 month storage period. This is consistent with previous observations showing the PICS bags have a more stable R.H. environment than other bag types [7, 21]. A stable moisture environment is beneficial from a farmer’s perspective, as it creates an expectation that initial grain moisture conditions will remain the same so long as the bag is closed. For tropical regions, having a barrier against water vapor transmission would prevent stored maize from absorbing water when humidity is high [22] and from losing water when it is low.

Overall, the oxygen and moisture conditions within PICS triple-layer bags appear to maintain the quality of stored maize. Both PICS bag treatments had similar germination rates to the non-infested woven bag treatment group and higher rates than the infested woven group. This agrees with previous observations for maize stored in PICS bags at similar moisture ranges [23].

In the present experiment, we did not find a significant difference in insect damage between triple and woven bags. This may have been a consequence of the relatively low levels of infestation used and other factors, such as cooler environmental temperatures in the later periods of the study. Even so, germination rates were significantly lower in infested woven bags versus triple bags. This effect is possibly due to the maize weevil adults and larvae destroying the germ in the seed at higher rates in woven bags than in PICS bags.

PICS bags are an effective method of controlling insects on maize for smallholder farmers. Not only is the damage inflicted by insects severely limited, but other values of grain quality are preserved. New insights, such as the buffering properties of the inter-liner space, improve our understanding of how PICS bags create low-oxygen environments.
